# Anxiety-like behaviour in mice after mild repetitive head impacts during the subacute phase

**DOI:** 10.3389/fnbeh.2026.1839876

**Published:** 2026-06-02

**Authors:** Andrej Durgala, Marian Horvath, Michaela Skrabanova, Martin Cente, Peter Filipcik

**Affiliations:** Laboratory of Molecular and Cellular Neurobiology, Institute of Neuroimmunology, Slovak Academy of Sciences, Bratislava, Slovakia

**Keywords:** anxiety, markerless pose estimation, mice, open field test, thigmotaxis, traumatic brain injury

## Abstract

Traumatic brain injury (TBI) is a major health concern in the current population. Animal models are increasingly important for identifying early neurobehavioural changes associated with mild to severe as well as repetitive brain injuries. In this study, we analysed behavioural changes in a mouse model of repetitive mild TBI using unbiased quantification metrics. Our approach enabled automated markerless pose estimation of thigmotaxis together with spatial working memory and locomotor activity assessments specifically during the scarcely-studied subacute phase after brain injury. We found that animals exposed to repetitive mild head impacts developed anxiety-like behaviour, manifested by avoidance of the central zone and increased thigmotaxis in the open field test, while maintaining total distance travelled compared to the control group. However, analysis of exploratory behaviour and spatial working memory in the Y-maze, along with assessment of sensorimotor coordination and balance using rotarod tests, revealed no significant difference between the TBI and control groups. These findings suggest that repetitive mTBI is associated with selective anxiety-like behaviour during the subacute phase after brain injury, without detectable motor or cognitive deficits, reflecting the early affective symptomatology frequently reported in clinical populations with mTBI.

## 1 Introduction

Traumatic brain injury (TBI) is a leading cause of injury-related mortality and long-term disability worldwide. It is commonly caused by falls, sports-related head impacts, motor vehicle accidents, blast exposures, or domestic violence (Guan et al., 2023; Butler et al., 2025). TBI is classified as mild, moderate, or severe based on the clinical severity and injury characteristics (Hawryluk and Manley, 2015). More broadly, TBI is recognized as a heterogeneous syndrome requiring multifaceted therapeutic strategies targeting various pathological processes simultaneously (Blennow et al., 2016). A subset of individuals who sustained mild TBI (mTBI) develop persistent symptoms. Notably, anxiety occurs in approximately 17% of TBI patients and is about twice as common compared to non-injured individuals (Dehbozorgi et al., 2024). Moreover, repetitive mTBI is associated with the increased risk of long-term emotional and behavioural sequelae (Howlett et al., 2022).

Research in human mTBI is complicated by the heterogeneity of injury mechanisms, clinical presentation, and individual susceptibility. Although the difference from human neuropathology is obvious, mouse TBI models provide several advantages, including standardised injury mechanisms, consistency in age, sex, genetic background, and reproducible injury parameters aligned with defined injury severities (Xiong et al., 2013). Nevertheless, behavioural findings in studies of repetitive mTBI remain inconsistent, in part due to the lack of standardised analytical methods and testing time points. The subacute period (>3 days and <3 months) represents a promising therapeutic window after mTBI, as pathological processes remain active and potentially amenable to intervention during this period. Characterization of the affective phenotypes present during this window is therefore a prerequisite for designing targeted interventions (Shultz et al., 2020; Tseitlin et al., 2025). The four-week post-injury time point falls within this subacute window and is biologically meaningful for several reasons. Secondary injury cascades, including neuroinflammation, microglial and astrocytic reactivity, glymphatic pathway dysfunction, blood–brain barrier disruption, and progressive tau hyperphosphorylation, remain active throughout this period and represent tractable targets for therapeutic intervention (Iliff et al., 2014; Loane and Kumar, 2016). Despite this, behavioural studies in mTBI models have predominantly clustered within the first week or beyond 3 months post-injury, leaving the subacute window comparatively underexplored (Shultz et al., 2020; Tucker and McCabe, 2021).

Given the inconsistent behavioural findings in models of repetitive mTBI, this study aimed to characterise anxiety-like behaviour, spatial working memory, and motoric performance using an unbiased quantification method. Our analytical approach enabled automated markerless pose estimation of thigmotaxis (DeepLabCut), a metric of anxiety-related open-field avoidance together with locomotor activity assessments. In addition, supportive measures assessing cognitive flexibility and sensorimotor functions in the subacute period after repetitive mTBI were also performed.

## 2 Methods

### 2.1 Animals

All mice used in this study were C57BL/6J (RRID:IMSR_JAX:000664). Animals were bred and maintained at an animal facility of the Institute of Neuroimmunology SAS. Six-month-old males were used. They were maintained under standard laboratory conditions (23 ± 1 °C, 50 ± 10% humidity, and 12-h light/dark cycle) with *ad libitum* access to food and water. Mice were randomly assigned to the 5xTBI (*n* = 6) and Sham group (*n* = 5). Given the exploratory nature of this study, sample sizes were selected based on prior repetitive mTBI literature employing comparable group sizes (Mouzon et al., 2012; Gold et al., 2018); effect sizes with confidence intervals are reported to enable power calculations for future confirmatory studies. All experiments were carried out in accordance with the approval of the Ethics Committee of the Institute of Neuroimmunology SAS and the State Veterinary and Food Administration of the Slovak Republic.

### 2.2 Injury induction protocol

Head impact was performed according to the modified protocol (Gold et al., 2018; Rubenstein et al., 2019). Animals were weighed before every surgical procedure. General anaesthesia was induced with isoflurane (3% in oxygen, 1.0 L/min, IsoFlo 100% w/w Inhalation Vapour, Zoetis Animal Health ApS, Belgium) and maintained via a nose cone (2% isoflurane in oxygen; 1.0 L/min). The mouse was placed prone on a polyurethane foam pad, and the head was stabilised by lightly taping the ears to the foam surface. The head was shaved to ensure direct skin contact, and an ophthalmic ointment was applied (Hypromeloza-P (5 mg/mL), Unimed Pharma, Slovak republic). The 5-mm-diameter metal probe tip was aligned midline to the sagittal suture, with its anterior edge positioned at a virtual line through the rearmost part of the eyes (approximately 1–2 mm posterior to bregma). The tip was then displaced 3 mm to the right of midline. The impact was induced at a speed of 5.0 m/s, 1.0 mm depth, and 100 ms dwell time. Head impact was produced using an electromagnetic impactor (Leica Impact One Stereotaxic Impactor, Leica Biosystems, Germany, RRID:SCR_025114). This protocol corresponds to mild injury severity according to established criteria (DeWitt et al., 2013; Mouzon et al., 2014). The total procedure took approximately 7 min per animal. To verify that each impact met the criteria for mild injury, we monitored three parameters: 1. A short period (<30 s) or absence of post-impact apnoea (recognized as an animal analogue to human loss of consciousness), 2. A short period (<6 min) of righting reflex (the time it takes for the mouse to right itself onto all four paws), 3. Negative signs of skull fracture at the time of euthanasia, and no history of major haemorrhage. Impacts were applied at 48-h intervals over five sessions, allowing for recovery from anaesthesia while modelling the cumulative effects of the injury (Mouzon et al., 2012). After the impact, the time to resume breathing (apnoea) was measured for each animal. The mouse was then removed from the device and allowed to recover in its home cage. The animal was placed in a supine position and the time required to roll over (righting time) was measured. No analgesia was used. Control (Sham) mice received the same procedure without the impact.

### 2.3 Behavioural assays

To maximize the validity of behavioural experiments and minimize animal distress, all animals were handled daily for 1 week prior to the start of behavioural testing. Behavioural testing was performed during the light cycle, and the observation and subsequent data analysis were performed blinded to treatment.

#### 2.3.1 Open field test

To assess locomotor activity, anxiety, and exploratory tendencies in a novel environment, an open field test (OFT) was performed (Sham *n* = 5; 5xTBI *n* = 6). Mice were transferred in their home cages to the behavioural testing room, which was pre-set with red light (10 lux), and allowed to habituate for approximately 30 min. Behaviour was recorded using a PointGrey Flea3 camera (1.3 MP Mono USB3 Vision, FL3-U3-13S2M-CS). Red light was used to reduce photic stress and approximate the animals’ active (dark) phase during light-cycle testing, thereby minimising ceiling effects in anxiety measures (Zhou et al., 2018; Tseitlin et al., 2023). The size of the custom-made open field arena was 45 × 45 × 45 cm, the central zone was defined as a 22.5 × 22.5 cm square centred in the arena; the peripheral zone comprised a 5 cm band adjacent to the wall. Each trial started when the animal was placed in the centre of the arena and allowed to move freely for 10 min (Drayson et al., 2023; Zhou et al., 2018). After recording, the mice were returned to their home cages. Between each trial, the arena was cleaned with Virkon S (Lanxess, Germany).

#### 2.3.2 Y-maze test

For assessment of spatial working memory, a Y-maze test was performed. The test relies on the animal’s natural tendency to explore novel environments, specifically by assessing how often they enter a previously unvisited arm and how many spontaneous alternations (consecutive entries into three different arms) were made. A custom-made Y-shaped maze consisted of three equal-length arms with dimensions of 36 × 8 × 20 cm (length × width × height) spaced 120° apart and open on top. The Y-maze test followed a modified protocol (Prieur and Jadavji, 2019; Wolf et al., 2016). The animal was placed directly in arm B, facing away from the centre, and allowed to move freely through the device for 8 min while being recorded. The spontaneous alternation percentage [(number of spontaneous alternations)/(total number of arm entries − 2) × 100] and total distance travelled were the primary Y-maze endpoints.

#### 2.3.3 Rotarod

Motor coordination was assessed 4 weeks after the last impact using an accelerating rotarod (Panlab, Harvard Apparatus), set to linearly accelerate from 4 to 40 rpm over 300 s, based on modified protocols (Scholz et al., 2015; Deacon, 2013). Animals from the same cage were placed in separate lanes on the rod that was initially rotating at 4 rpm (standby mode) and allowed to acclimatize for 1 min. The rod was then switched to acceleration mode. Mice underwent three training trials with a 10-min inter-trial interval; data from training trials were not recorded. After training, animals completed three test trials on the same day in which the latency to fall was measured and averaged across trials. If the animal clung to the rod and completed a full passive rotation, the timer was stopped, the event was noted, and the animal was returned to its home cage.

#### 2.3.4 Pose estimation and behavioural quantification

All videos from the OFT and Y-maze were analysed using DeepLabCut (DLC; version 2.3.9; RRID:SCR_021391; Mathis et al., 2018; Nath et al., 2019; Bogachev et al., 2023). DLC model parameters for the OFT and Y-maze are reported in Table 1. Multiple body points (nose, left ear, right ear, centre of body, tail base) were labelled.

**Table 1:** DeepLabCut pose estimation model and arena parameters for the open field test and Y-maze test.

Parameter	Open field test	Y-maze test
Video frame rate	120 FPS	120 FPS
Video resolution	1,328 × 1,048 px	1,328 × 1,048 px
Body points labelled	Nose, L ear, R ear, centre, tail base	Nose
Labelled frames (videos)	195 (11 videos)	440 (11 videos)
Frame extraction method	Uniform	Uniform
Train/test split	95%/5%	95%/5%
Network architecture	ResNet-50	ResNet-50
Training iterations	50,000	100,000
Batch size	8	8
Training error (raw/corrected)	3.29/2.9 px	2.76/2.76 px
Test error (raw/corrected)	7.19/4.44 px	11.46/11.57 px
Likelihood threshold	≥0.6	≥0.6
Arena dimensions	45 × 45 cm	36 × 8 × 20 cm (per arm)
Centre zone definition	22.5 × 22.5 cm (centred square)	8 cm equilateral triangle (between arms)
Session duration	10 min	8 min

Zone occupancy in the OFT (centre vs. periphery) was determined from the centre-of-body coordinate. Pixel coordinates were converted to centimetres using an arena calibration factor derived from the known arena side length. From the tracked trajectories, we computed the distance travelled (sum of frame-to-frame Euclidean displacements) and the elapsed time (difference in frame-to-frame occupancy). Distance and time indices were defined as the proportion of total trajectory length and total tracked time travelled/spent within the central or peripheral zone, respectively. In this study, thigmotaxis is operationally defined as the distance or time index in the peripheral zone. Within-session habituation was assessed by computing distance and time indices across consecutive one-minute intervals.

For the Y-maze, arm entries and exits were defined by the nose coordinate crossing predefined boundary lines at each arm’s junction with the central zone; spontaneous alternation was then computed from the resulting sequence of arm entries. All behavioural metrics were calculated using custom scripts and analysed blind to group labels until completion of pre-processing and endpoint extraction. Heat maps were derived from the tracking data with the OpenCV (v4.13.0.92, RRID:SCR_015526), NumPy (v2.4.2, RRID:SCR_008633), and Matplotlib (v3.10.8, RRID:SCR_008624) Python libraries.

### 2.4 Statistical analysis

All analyses were performed using Python (v3.11.9, RRID:SCR_008394) with the NumPy (v2.4.2, RRID:SCR_008633), Pandas (v3.0.0, RRID:SCR_018214), Scipy (v1.17.0, RRID:SCR_008058), Statsmodels (v0.14.6, RRID:SCR_016074), Matplotlib (v3.10.8, RRID:SCR_008624), Lifelines (v0.30.0, RRID:SCR_024899), and Pingouin (v0.5.5, RRID:SCR_022261) libraries. Normality of residuals was assessed and confirmed with the Shapiro–Wilk test, and group comparisons were performed using Welch’s *t*-test. Effect sizes (Hedges’ *g*) and 95% confidence intervals (CI) are reported to facilitate interpretation and future power calculations, given the exploratory nature and small sample size. Data are presented as mean with 95% CI, with a 2-tailed *p*-value <0.05 considered statistically significant. To explore the temporal profile of thigmotaxis during one-minute time intervals, we fitted a linear mixed-effects model with group, time, and their interaction as fixed effects and animal as a random intercept; this model was evaluated with a likelihood-ratio test (LRT). Statistical summary of behavioural outcomes is reported in Table 2.

**Table 2:** Statistical summary of behavioural outcomes.

Measure	Sham mean [95% CI]	5xTBI mean [95% CI]	Test	Stat.	df	*p*-value	Hedges’ *g* [95% CI]
Total OFT distance (cm)	2854.10 [2083.30, 3624.90]	2972.81 [2338.87, 3513.67]	Welch *t*	−0.21	7.70	0.8382	−0.12 [−1.61, 1.11]
OFT thigmotaxis distance index	0.47 [0.40, 0.57]	0.62 [0.54, 0.69]	Welch *t*^a^	−2.29	8.53	0.0496	−1.27 [−3.61, −0.27]
OFT thigmotaxis time index	0.54 [0.44, 0.67]	0.68 [0.58, 0.78]	Welch *t*^a^	−1.55	8.58	0.1582	−0.86 [−2.83, 0.17]
OFT central zone distance index	0.22 [0.17, 0.26]	0.13 [0.1, 0.17]	Welch *t*^a^	2.18	8.74	0.0208	1.55 [0.51, 5.46]
OFT central zone time index	0.2 [0.12, 0.26]	0.09 [0.05, 0.14]	Welch *t*^a^	2.01	8.13	0.0782	1.13 [0.07, 4.25]
Y-maze alternation	0.64 [0.59, 0.68]	0.57 [0.49, 0.65]	Welch *t*^a^	1.27	7.33	0.2434	0.66 [−0.31, 3.65]
Y-maze distance (cm)	1005.3 [895.2, 1097.1]	1163.3 [1044.9, 1273.7]	Welch *t*	−1.83	9.00	0.1010	−0.99 [−2.72, −0.02]
Rotarod latency (s)	89.8 [80.6, 99.4]	85.2 [70.2, 104.7]	Welch *t*	0.41	7.61	0.6905	0.22 [−0.75, 2.22]
Rotarod speed (rpm)	14.78 [13.67, 15.93]	14.22 [12.42, 16.46]	Welch *t*	0.41	7.61	0.6905	0.22 [−0.73, 2.22]

Sham *n* = 5, 5xTBI *n* = 6. *g* = Hedges’ *g* effect size.

a Welch’s *t*-test performed on logit-transformed data.

## 3 Results

**Figure 1:**
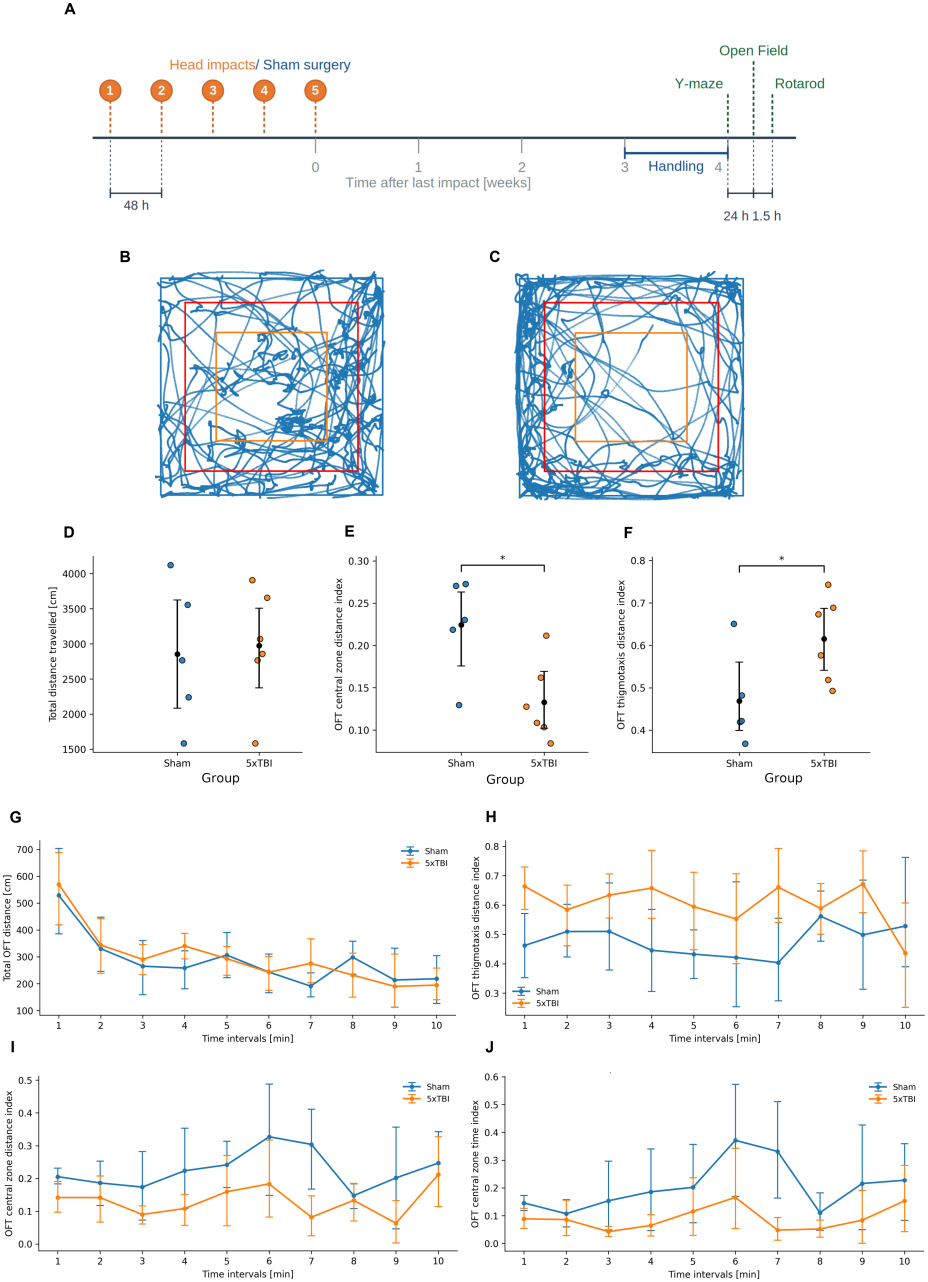
Study design and open field test outcomes after repetitive mild closed-head injury. Schematic illustration of the study protocol **(A)**. Representative trajectories of open field tests for sham control **(B)** and 5xTBI **(C)** animals with the boundaries of the central zone (orange square) and peripheral zone (red square) shown. Quantification of the total distance travelled (in cm) during the 10 min session revealed no difference between groups **(D)**, while the head-impacted animals showed a significantly decreased central zone distance index **(E)** and a significantly increased thigmotaxis index **(F)** compared to the control group. The effect of habituation is illustrated by the decreasing profile of total distance travelled in both sham control and 5xTBI animals **(G)**. However, the thigmotaxis is manifested by an increased distance index in the peripheral zone **(H)** along with a decreased distance index **(I)** and time index in the central zone **(J)** in animals with 5xTBI compared to controls. Dots and bars in the graphs indicate the mean with 95% CI. Sham *n* = 5, 5xTBI *n* = 6. An asterisk indicates a *p*-value <0.05. Statistical details are provided in Table 2.

In this study, we investigated behavioural changes in response to mild repetitive head impacts in mice during the subacute phase (Figure 1A). Repetitive mild closed-head impacts did not induce overt sickness behaviour during the test period. Post-impact recovery was rapid, with all animals regaining normal righting and spontaneous activity within minutes. Animals did not differ in righting times (Sham: 79.4 s, 95% CI 45.3–113.5; 5xTBI: 77.7 s, 95% CI 20.6–134.7; *p* = 0.947) or body weight (Sham: 25.4 g, 95% CI 22.4–28.4; 5xTBI: 27.5 g, 95% CI 24.7–30.3; *p* = 0.204) after the last injury. These measures were comparable between Sham and 5xTBI mice, with no group differences at any time point. No apnoea was observed during the experiment, and no mortality was recorded after the injury.

### 3.1 Repetitive mTBI is associated with increased thigmotaxis

As shown by the open field test trajectories for Sham (Figure 1B) and 5xTBI (Figure 1C) mice, impacted animals exhibited stronger anxiety-like behaviour, preferring the relatively protected walls and corners over the exposed centre. The total distance travelled during the entire session was similar in Sham and 5xTBI animals (Figure 1D). The procedure did not induce apparent hypo- or hyper-activity, whereas spatial patterning of the exploration revealed additional differences (Table 2). Repeatedly impacted animals avoided the central zone of the open field, as documented by a statistically significant decrease in the central zone distance index and a large effect size (Figure 1E). This centrophobic behaviour of impacted animals is consistent with significantly increased thigmotaxis compared to the control group (Figure 1F). Habituation analysis in the OFT showed a typical decreasing trend in total distance travelled during consecutive one-minute intervals, indicating that there was no significant difference between groups (Figure 1G). However, the wall-following behaviour diverged early in the session, with 5xTBI mice already displaying increased thigmotaxis compared to Sham mice within the first minute (Figure 1H). In accordance with this observation, impacted animals showed a consistent decrease in the central zone distance index (Figure 1I) and central zone time index (Figure 1J) across all time intervals assessed in the open field test. It should be noted that the central-zone time index showed the same direction of effect as the central-zone distance index, with a comparable large effect size (Hedges’ *g* = 1.13, 95% CI 0.07–4.25), but did not reach statistical significance (*p* = 0.0782, Table 2). The two parameters capture related but mechanistically distinct aspects of centre avoidance. The distance index is movement-based and reflects active exploration of the centre, whereas the time index is occupancy-based and reflects sustained presence. This pattern is consistent with the avoidance phenotype, in which animals briefly enter the centre but tend to rapidly retreat to the periphery.

### 3.2 Repetitive mTBI does not significantly affect working memory, locomotor activity or sensorimotor coordination

**Figure 2:**
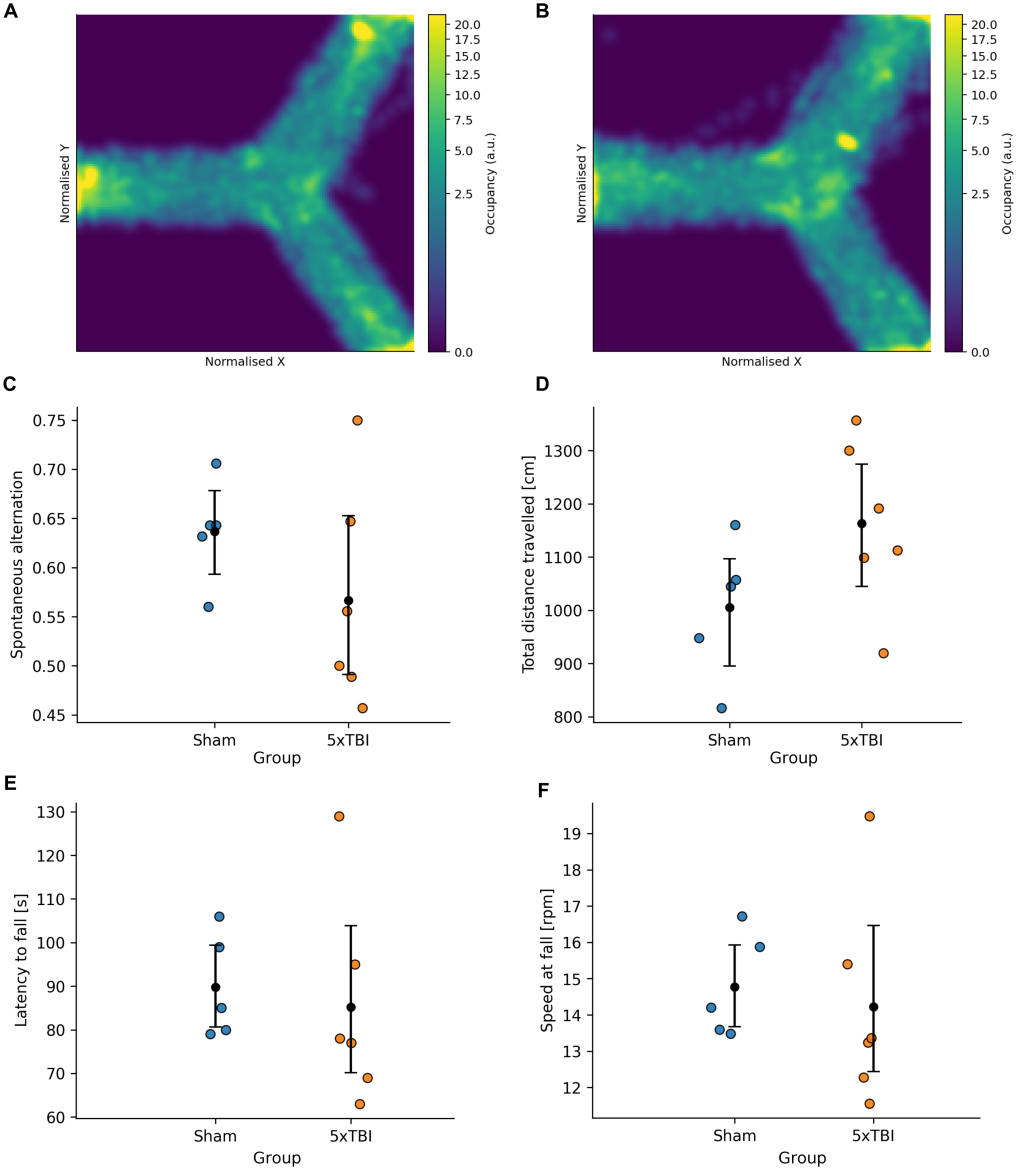
The effect of repetitive mTBI on working memory, locomotor activity, sensorimotor coordination, and balance. Heat maps tracking the spatial occupancy of sham **(A)** and 5xTBI **(B)** animals in the Y-maze. Quantification of spontaneous alternation of arms **(C)** and total distance travelled **(D)** by animals in the Y-maze revealed no difference between groups. Assessment of sensorimotor coordination and balance using the rotarod test showed no significant difference in latency to fall **(E)** or speed at fall **(F)** between groups. Black dots and bars in the graphs indicate the mean with 95% CI. Sham *n* = 5, 5xTBI *n* = 6. Statistical details are provided in Table 2.

Further behavioural analysis of the animals assessed spontaneous alternation and total distance travelled in the Y-maze test. Animals in both groups predominantly explored the maze periphery, frequently preferring the arms and avoiding the central intersection, indicating potential shelter-seeking behaviour. The presence of Sham and 5xTBI animals in Y-maze test is visualized at occupancy heat maps (Figures 2A,B). Spontaneous alternation in the Y-maze did not differ between Sham and 5xTBI groups, indicating no detectable impairment in short-term working memory after repetitive mild head impacts (Figure 2C; Table 2). Total distance travelled in the Y-maze was also comparable between groups (Figure 2D; Table 2). Assessment of sensorimotor coordination and balance using the acceleration rotarod test revealed no detectable differences in latency to fall (Figure 2E; Table 2) or speed at the time of fall (Figure. 2F; Table 2) in animals with 5xTBI compared to the control group. In summary, these data suggest that repetitive mTBI in our experimental setting did not detectably impair spatial working memory, exploratory behaviour, or locomotor activity during the subacute phase after brain injury.

## 4 Discussion

Along with the recent challenges in this field of research, our study aimed to investigate the subacute effects of repetitive TBI on behavioural parameters using unbiased methodological approach. We found that repetitive mild closed-head impacts induced a selective increase in thigmotaxis, an index of anxiety-like open-field avoidance, during the subacute phase (4 weeks) after brain injury. Furthermore, this phenotype was observed without concurrent deficits in locomotor activity, spatial working memory, or sensorimotor coordination. This behavioural profile is consistent with increased central-zone aversion, with animals entering the centre briefly but retreating rapidly to the periphery. Based on the natural tendency of rodents to avoid exposed areas, increased thigmotaxis is interpreted as an index of anxiety-like behaviour (Seibenhener and Wooten, 2015). A comparable dissociation between thigmotaxis and overall locomotor activity has been reported, with animals spending less time in the central zone after TBI, while total distance travelled remained unchanged (Beitchman et al., 2020). In our cohort, the dissociation between the statistically significant decrease in the central-zone distance index and the strong but non-significant decrease in the central-zone time index reflects a difference in statistical sensitivity rather than divergent biological signals. Both parameters show large effect sizes in the same direction, and the wider 95% confidence interval for the time index is consistent with the limited statistical power of an exploratory cohort of this size. Clinically, this pattern has a translational parallel, as mood and anxiety symptoms in patients with mTBI often occur without measurable cognitive or motor deficits (Howlett et al., 2022). However, it should be noted that a previous study using open field in a similar setting found no changes 3 weeks after single mild injury (Washington et al., 2012). Similarly, no change in the central zone distance was detected 30 days after three mild cortical impacts, although total distance travelled was increased in animals with TBI (Tucker et al., 2019). A reduction in central zone distance was observed within 1–20 days after a single severe impact, but not after single mild impact, indicating increased anxiety. However, other tests, such as the elevated zero maze, light–dark box, or marble burying test have shown conflicting results, rather suggesting a reduced anxiogenic effect of brain injury (Tucker et al., 2017). Assessment of anxiety outcomes in behavioural studies after brain injury appears to be time- and task-dependent. Increased anxiety was reported within the first month after a single moderate to severe TBI, as assessed in the elevated zero maze (EZM) and elevated plus maze (EPM), while anxiety-like behaviour decreased within 5–7 weeks, as measured in the OFT and EZM (Popovitz et al., 2019). The importance of test selection was illustrated in a study of mTBI, where neither the EPM nor the OFT detected anxiety-like changes 7 days after mTBI, whereas longer-duration paradigms (marble burying, light–dark box) revealed a difference between groups (Tseitlin et al., 2023). These findings indicate that the OFT, when used with appropriate parameters (arena size, lighting, session duration, and analytical endpoints), can detect anxiety-relevant behaviours in this model. However, we do not claim that the OFT is inherently superior to the EPM or EZM: prototypical anxiolytics fail to increase central-zone time in the OFT in C57BL/6J mice (Heredia et al., 2014; Thompson et al., 2015), and the EPM and EZM have yielded heterogeneous and often conflicting findings in the mTBI literature (Tucker and McCabe, 2021). All three open-arena assays have known limitations in sensitivity and predictive validity, and given the small cohort studied here, these data do not support any inference about their relative merits. Confirmation of the candidate phenotype described here will require independent cohorts assessed with a battery of complementary tests (e.g., light–dark box, marble burying, novelty-suppressed feeding).

Repetitive mTBI in our setting did not detectably impair short-term memory, exploratory behaviour, or locomotor activity assessed in the Y-maze during the subacute phase after injury, but the study was underpowered to detect anything smaller than a large effect. Similarly, no changes in spatial working memory were reported 2 weeks after mTBI, although Barnes maze testing at 1 month revealed learning and memory deficits (Cheng et al., 2014). Other groups have reported impaired cognitive function, spatial recognition and memory in comparable models (Balasubramanian et al., 2023; Bazaz et al., 2025). These heterogeneous data underscore the importance of assessing multiple cognitive domains and time points when characterising the behavioural consequences of head impacts.

The accelerating rotarod test revealed no detectable differences in motor coordination or balance between groups, consistent with the absence of vestibular injury and in line with the findings showing no motor deficits 1 month after the last injury (Gold et al., 2018; Tucker et al., 2019). Most reported motor deficits were present acutely, 1 day after three (Tucker et al., 2019) or ten repetitive head impacts (Gold et al., 2018). However, one study also reported reduced motor function even 3 weeks after triple TBI (Wu et al., 2022). Overall, these data suggest that the detection of motor deficits depends primarily on the injury model and early assessment period.

The subacute time point examined in this study (4 weeks post-injury) was selected based on converging biological and translational considerations. First, protein and gene expression changes in mTBI-affected brain tissue have been documented during this period, and exercise-based interventions have demonstrated therapeutic efficacy at this stage (Tseitlin et al., 2025, 2026). At this time point, glymphatic pathway dysfunction has been shown to persist for at least 1 month after experimental TBI and is mechanistically linked to the development of tau pathology (Iliff et al., 2014). In the subacute window, comparable repetitive mild closed-head models have demonstrated behavioural sensitivity without overt corpus callosum atrophy (Cheng et al., 2014; Mouzon et al., 2014; Gold et al., 2018). Similarly, this time point aligns with the earliest clinical assessment window for persistent post-concussion symptoms (Polinder et al., 2018). Finally, existing reviews of behavioural testing in TBI models confirm that most studies cluster within the first week or beyond 3 months post-injury (Shultz et al., 2020; Tucker and McCabe, 2021), while relatively few have characterised affective outcomes in this intermediate window. Whether the observed anxiety-like phenotype reflects these molecular changes remains to be determined.

In our experiments, we used a mild injury protocol that integrates elements from the controlled cortical impact and Marmarou acceleration models, allowing precise control of impact parameters without surgical intervention under brief general anaesthesia (Gold et al., 2018; Rubenstein et al., 2019). Behavioural phenotyping included markerless pose estimation based on DeepLabCut, which is not yet standard in mTBI research. Most studies comparable to our design used manual scoring or commercial tracking software (Tseitlin et al., 2023; Tucker et al., 2019). We do not claim that DLC improves the accuracy of zone occupancy estimates obtainable from established commercial tracking systems, which are also unbiased for this purpose. Rather, DLC provides an open-source, markerless, multi-keypoint, frame-wise tracking workflow whose pose-track files and analysis code can be inspected, audited, and re-analysed *post hoc* by other groups, supporting transparent reproduction and re-evaluation of archived video data without dependence on a specific commercial system. An additional design feature is the age of the cohort: most repetitive-mTBI mouse studies have been conducted in 2–4-month-old animals (Mouzon et al., 2012, 2014; Gold et al., 2018; Rubenstein et al., 2019), corresponding roughly to late adolescence or young adulthood. We instead used six-month-old males, an age that can be described as mature adult or early middle-aged. This age window complements the dominant young-adult mouse literature and may be relevant to adult-onset recreational, occupational, and accidental head injury. Behavioural and neuroinflammatory responses to TBI are known to be age-dependent (Tucker et al., 2017; Gold et al., 2018), so data from outside the canonical 2–4-month range are likely to complement rather than duplicate existing findings. Nevertheless, we acknowledge several limitations. The interpretation of our results is constrained by the absence of a female cohort, the small sample size, and a single time point of assessment. Males were chosen given the known confounding influence of the oestrous cycle on TBI-related behavioural outcomes and the divergent behaviour of stressed female rodents in the open field (Çevik et al., 2025; Shansky, 2018), which precludes generalisation of our findings to both sexes. Baseline testing was deliberately omitted because the OFT and Y-maze are one-trial novelty tests in which the central-exploration and spontaneous-alternation readouts habituate strongly on re-test and selectively attenuate the very anxiety- and exploration-relevant measures used here (Washington et al., 2012; Bourin, 2019; Tucker and McCabe, 2021). A within-subject pre-injury baseline would therefore have biased the post-injury comparison in a known direction, which, given the small cohort, could have masked a real injury effect or produced a spurious one. To partially compensate for the absence of within-animal anchors and the modest group size, we (i) used littermate Sham controls handled identically and tested in parallel, (ii) report Hedges’ g effect sizes with 95% confidence intervals alongside every *p*-value, and (iii) frame the entire study as exploratory and hypothesis-generating. Confirmation of these findings in an adequately powered cohort, with potential future arena-independent home-cage monitoring, will be required before firm conclusions can be drawn.

In conclusion, repetitive mild head impacts were associated with increased thigmotactic behaviour without detectable impairment of motor coordination or spatial working memory during the investigated subacute phase after brain injury. These findings are tentative and reflect the limited statistical power of an exploratory cohort; confirmation in adequately powered studies using complementary anxiety assays is required before broader inferences can be drawn. Within these constraints, the selective anxiety-like phenotype mirrors clinical observations that affective symptoms emerge acutely after mTBI (Zhang et al., 2024) and often occur independently of cognitive or motor recovery (Stein et al., 2019; Howlett et al., 2022; Levy et al., 2025). Together, these findings identify the subacute phase after repetitive mTBI as a time window characterised by a discrete affective phenotype, and provide a behavioural framework for future investigation of the molecular mechanisms underlying this stage of mTBI pathology.

## Data Availability

The raw data supporting the conclusions of this article will be made available by the authors, without undue reservation.
